# Kidney transplantation following iliac revascularization in severe atherosclerosis: a comparative study

**DOI:** 10.1007/s00423-023-02838-z

**Published:** 2023-02-25

**Authors:** Anna E. Cyrek, Lena Flögel, Arkadius Pacha, Moritz Kaths, Jürgen Treckmann, Andreas Paul, Maren Schulze

**Affiliations:** 1 Division of Vascular and Endovascular Surgery, Department of General, Visceral and Transplant Surgery, University Hospital Essen, University Duisburg-Essen, Essen, Germany; 2 Department of Urology, Evangelical Hospital Witten gGmbH, Witten, Germany; 3 Institute of Pharmacology and Toxicology, Ruhr-University Bochum, Bochum, Germany; 4 Department of General, Visceral and Transplant Surgery, University Hospital Essen, University Duisburg-Essen, Essen, Germany

**Keywords:** Iliac revascularization, Kidney transplantation, Outcome, Graft survival

## Abstract

**Background:**

Kidney transplantation (KT) has become the standard of care for patients with end-stage renal disease. However, as atherosclerosis progresses with time on dialysis, it causes increasing difficulties in implanting the graft. This is a comparative study analyzing complications and graft survival of recipients with iliac revascularization before transplantation.

**Methods:**

Between January 2006 and December 2015, 1691 kidney transplants were performed at our institution. We retrospectively analyzed eighteen patients with peripheral arterial disease (PAD) with the necessity of vascular revascularization before kidney transplantation to protect the inflow to the renal graft and to optimizing blood supply to the extremities. The primary endpoint included patient survival and graft survival. The secondary endpoints evaluate perioperative and early postoperative complication rates after kidney transplantation.

**Results:**

All patients enrolled in this study underwent two consecutive surgical procedures. No patient reported limb loss, and there was no additional perioperative morbidity or mortality related to the vascular procedure. Primary endpoints such as graft survival without dialysis and overall patient survival show 1-month survival of 100%, 1-year survival of 94.1%, and 5-year survival of 84.70%, respectively. One graft failure occurred 8 months after transplantation due to acute rejection, and there were two deaths over follow-up period due to myocardial infarction.

**Conclusions:**

Vascular repair before kidney transplantation is safe, and results are suggestive that it prolongs graft survival. These promising results should encourage other centers to address vascular repair before the transplantation to optimize blood supply to the extremity and the future graft. Although, the interpretation of our results must be cautiously because of the small and heterogeneous sample size, and the limitations of retrospective study design. Prospective trials with larger study populations are needed to confirm the results of this study and to identify significant differences.

## Introduction

The number of patients with chronic kidney disease (CKD) has been increasing over the last decade [1]. Chronic renal failure and hemodialysis are associated with hypertension and lipid disorders that predispose to accelerated atherosclerosis [2, 3]. Furthermore, atherosclerotic lesions in the aorto-iliac segment frequently occur in patients who are awaiting renal transplantation and become more common with increasing age and time on dialysis [4]. The success of organ transplantation depends on the quality of vascular access and anastomoses. Since time to transplant requiring dialysis is increasing, the incidence of aorto-iliac lesions will equally increase, [5] making the transplant increasingly difficult, including the risk of graft loss. Even though it is proven that survival in patients with PAD is significantly prolonged after kidney transplantation, [6] which can be underlined by the finding that arterial stiffness improves after kidney transplantation, [7] some transplant centers decline to accept patients with severe PAD, fearing an increase of graft losses, with consequences for their program [8].

Besides patients refusing access to kidney transplantation, the only other option is to review the operative strategy defining indications for aorto-iliac reconstruction before or at the time of transplantation, [9, 10] to improve blood supply to the extremities and to the graft. However, the optimal strategy for the timing and management of the vascular revascularization is still unknown.

The aim of our retrospective observational single-center study was to evaluate graft function and graft survival as well as intra- and postoperative complication rates of kidney transplant recipients with peripheral arterial disease (PAD) where vascular revascularization was performed before kidney transplantation over 10 years.

## Materials and methods

### Study population

This study was a single-center retrospective observational study of 1691 consecutive patients undergoing deceased donor kidney transplantation between January 2006 and December 2015. We analyzed patient charts of all kidney transplant recipients to identify those patients with peripheral artery disease (PAD), diabetes mellitus, hypertension, history of coronary artery disease, and smoking. Other data, including clinical presentation, operative details, graft, and patient survival, were extracted from medical records.

All patients receiving renal transplants were assessed preoperatively according to an established protocol that included a complete assessment of their vascular status based on medical history and physical examination. When atherosclerosis of the aorta, iliac, or femoral arteries was suspected, ankle-brachial pressure index (ABI) was measured, and axial computed tomographic scan (CT) of abdomen and pelvis was performed in case the ankle-brachial pressure index measurement detected any abnormality. The ABI measurements were grouped into four categories: normal (0.90–1.39), mild disease (0.70–0.89), moderate disease (0.40–0.69), and severe disease (0–0.39). Additionally, we analyzed the type of PAD presentation (claudication, chronical limb ischemia, ulceration/gangrene) and the level of PAD (stenosis, occlusion). Consequently, the indication for vascular reconstruction was defined by the transplant team and was based on vascular calcification, considering the risk of technical obstacles for renal transplantation caused by significant occlusive lesions. Patients with diffuse aorto-iliac atherosclerosis, type-C or -D aorto-iliac disease according to the Trans-Atlantic Inter-Society Consensus Document (TASC), [11] were considered as candidates for pretransplant vascular bypass surgery. Symptomatic patients with TASC type-A or -B aorto-iliac disease were considered as candidates for pretransplant iliac artery angioplasty. Patch angioplasty was performed in selected patients with extensive calcified plaques or a small iliac artery. Patients with normal ABI and without pronounced calcification of the iliac arteries were not revascularized and excluded from the study. Also patients undergoing re-transplants, younger than 18 years and those with missing data were also excluded from the study.

We retrospectively analyzed patients with the necessity of vascular revascularization before kidney transplantation to protect the inflow to the renal graft and to optimizing blood supply to the extremities. Finally, data of 18 patients with vascular revascularization were available for complete analysis. The primary endpoint included patient survival and graft survival. The secondary endpoints evaluate perioperative and early postoperative complication rates after kidney transplantation.

All study procedures adhered to the Declaration of Helsinki and were approved by the Institutional Ethics Committee (18–8023-BO). All patients provided written informed consent for kidney transplantation.

### Surgical procedures

All procedures were performed with general endotracheal anesthesia with continuous radial artery pressure monitoring and all recipients undergoing deceased donor kidney transplantation. Patients underwent kidney transplantation into the right or the left iliac fossa using an extraperitoneal approach. The vein was anastomosed to the external iliac vein in end-to-side manner. End-to-side anastomosis of renal artery was done to the external iliac artery. Lower pole arteries were always reconstructed and connected to arterial flow. Accessory arteries were reimplanted to the main renal artery in an end-to-side manner. Sutures of Prolene (6/0 or 7/0), (Ethicon, Germany) were used for vascular anastomoses. The ureter was reimplanted to the urinary bladder in standard Lich-Gregoir manner in all cases. A double-J stent (C. R. Bard GmbH, Karlsruhe, Germany) was routinely inserted into the ureter and removed 6 weeks after transplantation.

All patients received triple-immunosuppression with calcineurin inhibitors, such as cyclosporine or tacrolimus, antimetabolic agents, and low-dose steroids. Perioperative antibiotic prophylaxis was a second-generation cephalosporin used routinely for patients undergoing simple renal transplantation or for those who had a prosthetic replacement. At our institution, intraoperative heparin is not given as intravenous, and, postoperatively, there was only prophylactic anticoagulation therapy.

### Postoperative assessment

Postoperatively, patients were monitored for clinical (blood pressure, urine output), biological (ionic balance, serum creatinine), and sonographic signs of complications at defined time points. Graft function was initially evaluated by urine production within the first 24 h after transplantation and by serum creatinine levels at defined time points.

The presence of delayed graft function (DGF) was defined as the need for dialysis within the first week after surgery and/or failure of creatinine clearance to rise above 10 mL/min within the first 5 postoperative days irrespective of dialysis need, acute rejection, and immunosuppressant regimen at discharge.

Any complication or need for intervention following surgery was noted. Postoperative complications were rated according to the Clavien-Dindo classification [12].

Follow-up data were collected by our transplant program in cooperation with the Department of Nephrology of the University of Essen, Germany. Initially, patients were evaluated every 2 weeks until 3 months and every month thereafter until 6 months. After 6 months, the follow-up examination carried out every 3 months.

### Statistical analysis

Statistical analysis was performed using IBM SPSS Statistics version 24 (SPSS, Chicago, Ill). Graphs were created using SigmaPlot for Windows version 10.0 (Systat Software GmbH, Erkrath, Germany). Data were presented as mean (standard deviation) or median (range) as appropriate.

## Results

A total of 1691 patients underwent deceased kidney transplantation between January 2006 and December 2015 at our institution. According to the inclusion and exclusion criteria, data of 18 patients (9 males, 50%; and 9 females, 50%) with vascular revascularization were available for complete analysis. The reason for revascularization was, in all cases, iliac occlusive disease. These patients had multiple comorbid conditions including diabetes (*n* = 7), ever smoker (*n* = 8), coronary artery disease (*n* = 9), and hypertension (*n* = 14). The mean age of patients was 51.56 + / − 10.77 years. The indication for renal transplantation in all these patients was the progression of chronic renal insufficiency requiring long-term dialysis. All were recipients of their first kidney transplant, and in these patients, the artery of the transplanted kidney was implanted on the revascularization side. Baseline characteristics are presented in Table 1. The mean follow up period until December 2018 was 114 months (± 67).

**Table 1:** Baseline characteristics of 18 PAD patients with iliac revascularization included the final analysis

Characteristic	Patients with revascularisation
	*n* = 18
Age, years, mean (SD)	51.56 ± 10.77
Men, no. (%)	9 (50%)
Donor type, no. (%)	
Deceased	18 (100%)
Graft failure, no. (%)	2 (11.11%)
Cause of end-stage renal disease	
Diabetis mellitus, no. (%)	7 (38.89%)
Ever smoker, no. (%)	8 (44.44%)
Coronary artery disease, no. (%)	9 (50%)
Hypertension, no. (%)	14 (77.78%)
Peripheral arterial disease (PAD)	18 (100%)
by ankle-brachial-index (ABI)	
Low ABI	
Severe: 0–0.39	4 (22.22%)
Moderate: 0.40–0.69	12 (66.67%)
Mild: 0.70–0.89	2 (11.11%)
Medical history	
Glomerulonephritis	3 (16.67%)
Diabetic nephropathy	2 (11.11%)
Familial cystic kidneys	2 (11.11%)
Hypertensive nephropathy	2 (11.11%)
Interstitial nephritis	2 (11.11%)
Malformations	2 (11.11%)
Others	2 (11.11%)
Not known	3 (16.67%)
Vasculitides	0 (0%)
Type of revascularization	
PTFE vascular graft	3 (16.67%)
Cryopreserved vascular vessel	2 (11.11%)
Stent implantation (common iliac artery)	4 (22.22%)
Transluminal angioplasty (common iliac artery)	3 (16.67%)
Endarterectomy with bovine pericardium patch (common iliac artery)	2 (11.11%)
Angioplasty with stent implantation (common iliac artery)	2 (11.11%)
Aorto-femoral bypass	1 (5.56%)
Aorto-bifemoral bypass	1 (5.56%)

In accordance with our selection criteria, all patients with iliac repair received a non-contrast pelvic CT, which identified significant calcifications of the arterial system. The decision to perform a vascular reconstruction was made by an interdisciplinary transplant team to protect the inflow to the renal graft and to optimizing blood supply to the extremities before kidney transplantation. Vascular replacement in patients occurred 7.78 months (range, 1 to 21 months) before kidney transplantation, and all patients underwent elective repair. For this purpose, in five patients (27.78%), vascular prostheses were implanted (3 × a PTFE vascular graft, a 2 × cryopreserved iliac vessel), and in four patients (22.22%), common iliac arteries were stented. In three cases, stenosis of the iliac vessels was expanded using a transluminal angioplasty (16.67%); in two patients, endarterectomy procedures with bovine pericardium (Vascu-Guard®, Saint Paul, MN) patch (11,11%) were performed; and, in two patients, angioplasty along with a stent implantation (11.11%) of the common iliac artery was conducted. Furthermore, one patient received an aorto-femoral bypass and one an aorto-bifemoral bypass (5.56%) (Table 1). Of note, all vascular reconstructions remained patent, and no patient reported limb loss. No patient developed signs of arterial graft infection or stenosis, and there was no need for secondary interventions during the follow-up period. Additionally, there was no perioperative morbidity or mortality related to the additional vascular procedures.

### Postoperative kidney function

**Fig. 1:**
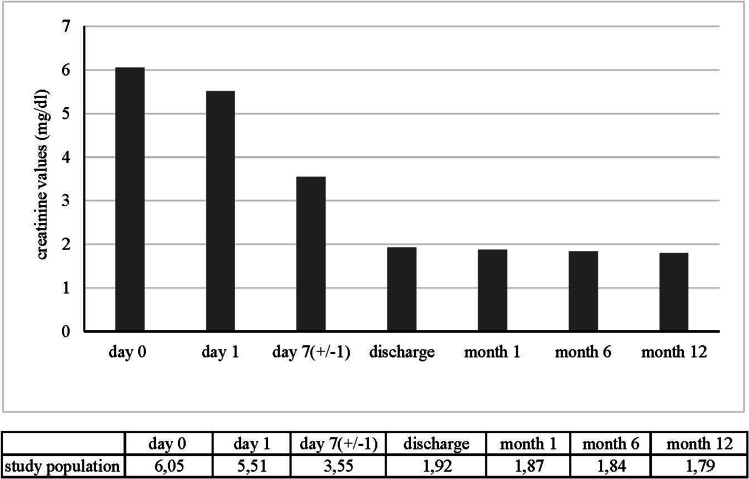
The course of the postoperative mean creatinine values in mg/dL

Kidney function was evaluated with a focus on primary function indicated through urinary production during the first 24 h. We evaluated levels of serum creatinine on day 1, two and seven after surgery, on the day of discharge, 1 month, 6 months, and, finally, 12 months following transplantation. Creatinine values over time are demonstrated in Fig. 1. As expected, there was a postoperative decrease in serum creatinine concentration, and none of the patients had a significant transient worsening of renal function. Mean serum creatinine on discharge was 1.92 mg/dL (range 0.88–5.06 mg/dL) in our cases.

### Complications

**Fig. 2:**
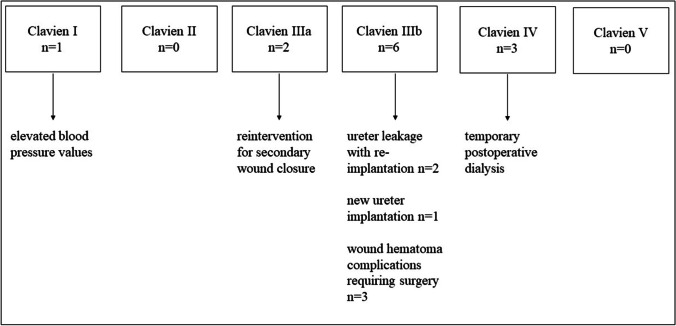
Distribution of postoperative complications using the Clavien-Dindo classification

Six patients (33.33%) had one or more postoperative complications. One patient (5.56%) had elevated blood pressure values and thus a type-I complication during postoperative course. Two patients (11.11%) had type-IIIa complications (secondary wound closure without general anesthesia, partly using VAC therapy). A total of six type-IIIb complications were documented, such as wound complications requiring surgery, ureter leakage with reimplantation and revisions in intubation anesthesia, new ureter implantations, and/or hematoma clearance under general anesthesia in three different patients (16.67%). Type IVa complications occurred in three patients. All of them required temporary, postoperative dialysis (16.67%). Details of complications according to Clavien-Dino classification are demonstrated in Fig. 2.

### Graft and patient survival

Regarding the documented endpoints, one patient (5.56%) was dialyzed again 82 months after kidney transplantation during follow-up. In another patient (5.56%), the kidney graft had to be removed 8 months following implantation caused by rejection, and the same patient died 29 months after the original transplant. Another patient died 44 months after kidney transplantation. Thus, a total of two deaths (11.11%) were recorded because of myocardial infarction.

Graft survival without dialysis and overall patient survival show 1-month survival of 100%, 1-year survival of 94.1%, and 5-year survival of 84.70%, respectively. All survivor recipients have stable renal function in follow-up period. No patient developed signs of arterial infection or stenosis, and there was no need for secondary interventions.

## Discussion

Patients with severe atherosclerosis account for 5.9% of all kidney transplant recipients (3.49%, our patient collective). Selection of arterial anastomotic site and method of arterial anastomosis by careful preoperative and intraoperative examination are extremely important [13]. As reported previously, only 0.7–3.6% of the recipients undergo aorto-iliac reconstruction before, during, or after renal transplantation [10, 14–17]. However, studies on aorto-iliac reconstruction before kidney transplantation are limited, and the most suitable timing for vascular reconstruction of aorto-iliac vessels in renal recipients has been discussed controversial [18]. Nevertheless, time to transplant requiring dialysis is increasing and the incidence of aorto-iliac lesions [5]. To avoid a higher rate of graft losses, some centers rather decline patients with severe PAD than consider surgical revascularization before transplant. In our opinion, these procedures are safe, could be performed as an elective procedure, and could optimize blood supply to the extremities and to the graft.

In our study, the total number of patients with PAD and CKD was 59 (3.5%). However, only 18 patients (1.1%) underwent iliac revascularization before kidney transplantation. This demonstrates that only a limited number of patients with PAD and CKD received vascular revascularization before kidney transplantation. Furthermore, the optimal strategy for the timing of the aortic disease revascularization and kidney transplantation is unknown. Severe deterioration in the vascular status of patients listed for transplantation may be due to longer waiting times. Thereby, severe arteriosclerosis and peripheral arterial occlusive disease can represent contraindi-cations for kidney transplantation in rare cases. In our center, an extensive workup of the vascular status of patients is performed before putting them on the waiting list. Every patient is investigated by ABI and duplex sonography. Additionally, according to clinical symptoms and risk factors, we performed angiography, MR angiography, and CT scans. During the evaluation, we collected sufficient information about the peripheral vascular status to correct critical stenosis by interventional or operative therapy, as needed. For this reason, we rarely excluded patients for kidney transplantation (approximately 1–2 patients/year) because of extensive arteriosclerosis. Moreover, unintended simultaneous revascularization was extremely rare (3 patients) due to dissection of the intima following clamping.

Postoperative kidney function showed a postoperative decrease in serum creatinine concentration, and none of the patients had a significant transient worsening of renal function. In the literature, postoperative kidney function after aorto-iliac repair based on creatinine values is described only by Pittaluga et al., demonstrating values of below 200 µmol/L (< 2.26 mg /dL) in 27 patients (87.1%) 1 month after kidney transplantation [18]. Comparable values in our study at the time of discharge were on average 1.92 mg/dL. Direct comparison of these two studies is difficult because Pittaluga et al. did not give average values but only the proportion of patients in relation to the limit of 200 µmol /L.

Primary endpoints such as graft survival without dialysis and overall patient survival show 1-month survival of 100%, 1-year survival of 94.1%, and 5-year survival of 84.70%, respectively. The interpretation of our results must be cautiously because of the small and heterogeneous sample size of study population. Early graft failure in previous studies ranges from 8 to 11% [6, 19]. Loss of the only one kidney in our series was caused by acute rejection, not renal ischemia.

The Clavien-Dindo classification is applied as a simple tool to assess and report postoperative complications. However, this classification only categorizes postoperative complications into five grades, disregarding preexisting conditions and comorbidities. We registered complications in 66.66% (*n* = 12) of our patients. Pittaluga et al. [18] described 18 complications in 13 patients (36.1%) after kidney transplantation and previous vascular replacement, though a distinction between the individual types of complications based on the Clavien-Dindo classification was not made.

Matia et al. [17] also describe complications, but not categorized into Clavien-Dindo classification. This study also does not show what exactly was considered as a complication and whether the complications mentioned occurred in different patients or whether one or more patients had more than one complication, so no statement can be made about the overall complication rate.

Patients with CKD who have PAD are at higher risk of cardiovascular mortality, [20–23] and coronary artery disease is the major cause of death in patients with CKD before and after their transplants [24]. This is supported by the fact that survival in patients with PAD is significantly prolonged after kidney transplantation, [6] which can, additionally, be underlined by the finding that arterial stiffness improves after kidney transplantation [7]. In our study, perioperative mortality was not related to the additional vascular procedure. The 30-day mortality rate in all patients was 0, and all-cause mortality was 11.11%. These findings are comparable to those previously reported in the literature [25]. However, it would be worth considering whether simultaneous revascularization would lead to reduce the surgical risk. Although the sample size in the present study is small, on the basis of our experience, acceptable outcomes were attained of a “two-step approach.”

Based on our findings and other reports in the literature, we can state that vascular repair before kidney transplantation is safe, and results are suggestive that it prolongs graft survival. These promising results should encourage other centers to address vascular repair before the transplantation to optimize blood supply to the extremity and the future graft. Even though patients undergo two surgical procedures implicating postoperative complications, this strategy will increase overall patient survival and minimize graft loss. Optimal management of these patients is unknown. However, this study reflects our experience with this special patient population.

## Limitation

The greatest limitation of this study’s results is the rarity that patients receive vascular revascularization before the kidney transplant. As a result, only a small number of patients could be included. The fact that all the indications for surgery were discussed by the interdisciplinary transplant team limits the selection bias to a certain extent.

A comparison between a reconstruction group and a non-reconstruction group would likely be biased due to the different extent of PAD as non-reconstructive recipients had a mild ABI and fewer calcification than reconstructive patients did. For this reason, patients without vascular reconstruction or patients with emergency one-stage approach were not included in the further analysis.

## Conclusion

The patient collective used in the present study is small. However, on the basis of our experience, acceptable outcomes were attained. Although our results must be cautiously interpreted because of the small and heterogeneous sample size, and the limitations of retrospective study design, there is a clear trend that graft and in particular patient survival are superior if PAD patients receive iliac vascular revascularization before kidney transplantation. Prospective trials with larger study populations are needed to confirm the results of this study and to identify significant differences.

## Data Availability

The authors confirm that the data supporting the findings of this study could be requested from the corresponding author.
